# Combine, assign or delete? How to resolve different levels of taxonomic identification in chironomid datasets

**DOI:** 10.1007/s10933-026-00387-1

**Published:** 2026-03-30

**Authors:** Oliver Heiri, Stefan Engels

**Affiliations:** 1 Geoecology, Department of Environmental Sciences, University of Basel, Basel, Switzerland; 2 Department of Geography, Royal Holloway University of London, Egham, UK

**Keywords:** Microfossils, Lake sediment, Biotic assemblages, Taxonomic resolution, Reconstruction, Chironomidae

## Abstract

The analysis of fossil biotic remains in lake sediment records allows for the reconstruction of past ecosystem dynamics and as such forms a powerful tool for understanding ecosystem processes and environmental change on a range of spatial and temporal scales. However, when producing palaeoecological datasets, analysts are often not able to assign all specimens to morphotypes at the highest taxonomic resolution. As a result, datasets containing unprocessed or raw counts usually include categories of identification across multiple different taxonomic levels (e.g. species morphotype, genus, tribe or even family level), also for fossil remains that may actually have originated from the same taxon (e.g. the same species or species morphotype). Whilst different strategies to deal with this problem have emerged over the years, it is rarely described in papers how analysts dealt with this issue, and how datasets were processed from raw counts to a final dataset used for palaeoecological interpretation, numerical analysis or quantitative inference. Using chironomid identifications as an example, we here describe the four main strategies for dealing with such multi-level identifications that can be applied to unprocessed count data with different levels of taxonomic detail: combining, retaining, deleting and assigning. We discuss advantages and disadvantages of each of these strategies and illustrate their impacts on palaeoecological analyses using both theoretical and practical examples. We conclude that there is no one optimal way to deal with the issue of incomplete or multi-level taxonomic identifications of fossils that may have originated from the same taxon, but that analysts will have to determine the strategy that best befits their project on a case-by-case basis, taking into account the type and quality of data they are working with, as well as the overall aims of their research project. We recommend that the choice of strategy (or combined strategies) is clearly described in the form of a brief statement in the Methods section of manuscripts where primary data are presented and that ideally both the unprocessed raw count data and the processed data used for ecological and numerical analyses are archived where publication outlets allow for this. Whilst we use chironomid research as the primary example throughout this manuscript, the proposed strategies and recommendations are relevant to a wide range of microfossil groups.

**Supplementary Information:**

The online version contains supplementary material available at 10.1007/s10933-026-00387-1.

## Introduction

Biotic remains in lake sediment records provide information on past occurrences and abundances of specific indicator species and indicator groups in lakes and their surroundings. The analyses of such remains can be used to reconstruct past changes in ecosystem states (e.g. Bennion et al. 2014; Płóciennik et al. 2020; Perret-Gentil et al. 2024; Lapellegerie et al. 2026), response and recovery of lake ecosystems to disturbances (e.g. Pillsbury et al. 2021), and variations in species diversity in time (e.g. Smol et al. 2005; Engels et al. 2020). Indirectly, via species—environment calibration datasets, and numerical inference models (transfer functions) developed from these, such analyses can also provide quantitative estimates of past changes in environmental variables such as temperature (Lemmen and Lacourse 2018; Szabó et al. 2024), nutrient concentrations (Luoto 2011; Moyle et al. 2024), or lake water depth (Ni et al. 2023). Many of the studies aiming at reconstructing the presence, abundance and ecological relevance of particular species and species groups based on lake sediment records have focused on identifying morphological remains (microfossils) in the sediments. Together with geochemical (Bechtel and Schubert 2009; Butz et al. 2017), and more recently also molecular approaches (Domaizon et al. 2017; Edwards 2020; Blattner et al. 2025), these analyses have provided the bulk of our knowledge on past biotic communities in lakes and how these have changed on decadal to millennial time scales in response to past environmental changes.

Approaches using microfossils to reconstruct past ecological and environmental changes rely on identifying preserved structures of selected indicator groups under the microscope and assigning these to specific taxonomic groups, ideally at species or species group level. The taxonomic resolution of such analyses may differ for different structures of the same organism group preserved in the sediments, but importantly it may also differ depending on the depositional environment and the preservation state of the fossils that are examined. As a consequence, palaeoecological analyses may result in datasets where the same species or species group may have been identified to different taxonomic levels. For example, it is common that some remains in a sample can be reliably identified to the most detailed taxonomic level achievable (e.g. to species or species morphotypes), whereas others can only be assigned to the level of genera or subfamilies. Similarly, some specimens might not be reliably distinguished from morphologically very similar, related groups. Although most microfossil groups will be affected by this problem, some indicator groups, and some subgroups within certain indicators, may be particularly susceptible to iden tification issues. This specifically applies to groups which may fragment during deposition, fossilisation and sample preparation. The problem of incomplete preservation or of obscured morphological features hampering identification to the most detailed possible level applies to many biotic proxies encountered in lake sediment records, including but not limited to chrysophytes, cladocerans, diatoms, ostracods, *Pediastrum* and pollen. It also particularly applies to pal- aeoecological studies of chironomid subfossils. Here we focus on this group that is widely used in palaeoecological and palaeolimnological research. We specifically examine the tribe Tanytarsini within the Chironomidae as an example, as Tanytarsini remains are common in lake sediment records and are often identified to different levels of taxonomic detail depending on their preservation. However, most of our considerations and recommendations are valid for other groups within the Chironomidae and could also be applied to other microfossil groups routinely identified in lake sediment records.

When analysing their data, palaeoecologists carrying out microscopic analysis will have to decide on how to deal with different levels of taxonomic resolution within their datasets. Different analysts and schools of research have developed various strategies on how to resolve this problem. Strategies that have been used include retaining taxonomic groups that originate from the same species or species group as separate taxa, the amalgamation of categories at the coarsest taxonomic level (i.e. at a lowest taxonomic resolution), the deletion of coarser taxonomic groups not representing the highest achievable level of identification from the analysis, or assigning less reliably identified specimens to different, better-identified categories based on the proportion of specimens identified to the most detailed taxonomic level in the same sample. A number of studies are available that address the effects of the overall taxonomic resolution of microfossil analyses on palaeoecological interpretations (e.g. Velle et al. 2010) or on the results of numerical analyses based on palaeoecological data (e.g. Heiri and Lotter 2010). Others discuss decisions and merging of taxa necessary when combining and taxonomically harmonising palaeoecological datasets of different taxonomic resolution (e.g. Potapova et al. 2022, Flantua et al. 2023). However, how specimens identified to different taxonomic levels are dealt with within the same dataset during ecological and numerical interpretation has rarely or only very briefly been described in papers presenting chironomid or other microfossil data. As a consequence, it often remains unclear which strategy has been used for a given dataset, and researchers new to palaeoecological research (or microfossil identification in general) often do not recognize the relevance of this issue for their projects.

Here we describe the main strategies that can be used to deal with the problem of fossils of the same taxon identified to different taxonomic levels. We illustrate advantages and drawbacks associated with the different strategies based on both theoretical considerations and numerical simulations of artificial count data, and illustrate the impacts of each strategy on a recently published chironomid record from a lake sequence in Germany. We present and discuss one possible way of assigning specimens identified to coarser taxonomic groups to categories that have been identified to the highest possible taxonomic resolution, to illustrate some of the decisions that will have to be taken when applying this approach that has not previously been described in detail in the chironomid literature. Finally, we discuss issues and strategies that researchers may want to consider when designing their own solution to this problem and provide recommendations on how analysts should report on how they dealt with this issue when publishing microfossil count data.

## Multi-level taxonomic resolution in subfossil chironomid datasets

The Chironomidae are a widely distributed group of dipteran insects with an aquatic larval stage (Armitage et al. 1995). Compared to many other aquatic insect groups, remains of chironomid larvae preserve exceptionally well as fossils in lake sediments over thousands to hundreds of thousand years. Fossil chironomid remains have been analysed to reconstruct past lake ecosystem dynamics, early and twentieth century human impact, and the response of lake ecosystems to changes in oxygen (Quinlan and Smol 2001), pH (Belle and Johnson 2024), salinity (Dickson et al. 2014) or water depth (Nazarova et al. 2017). Particularly the reconstruction of past changes in temperature has received a considerable amount of attention in fossil chironomid research (Walker et al. 1997; Medeiros et al. 2012).

**Fig. 1:**
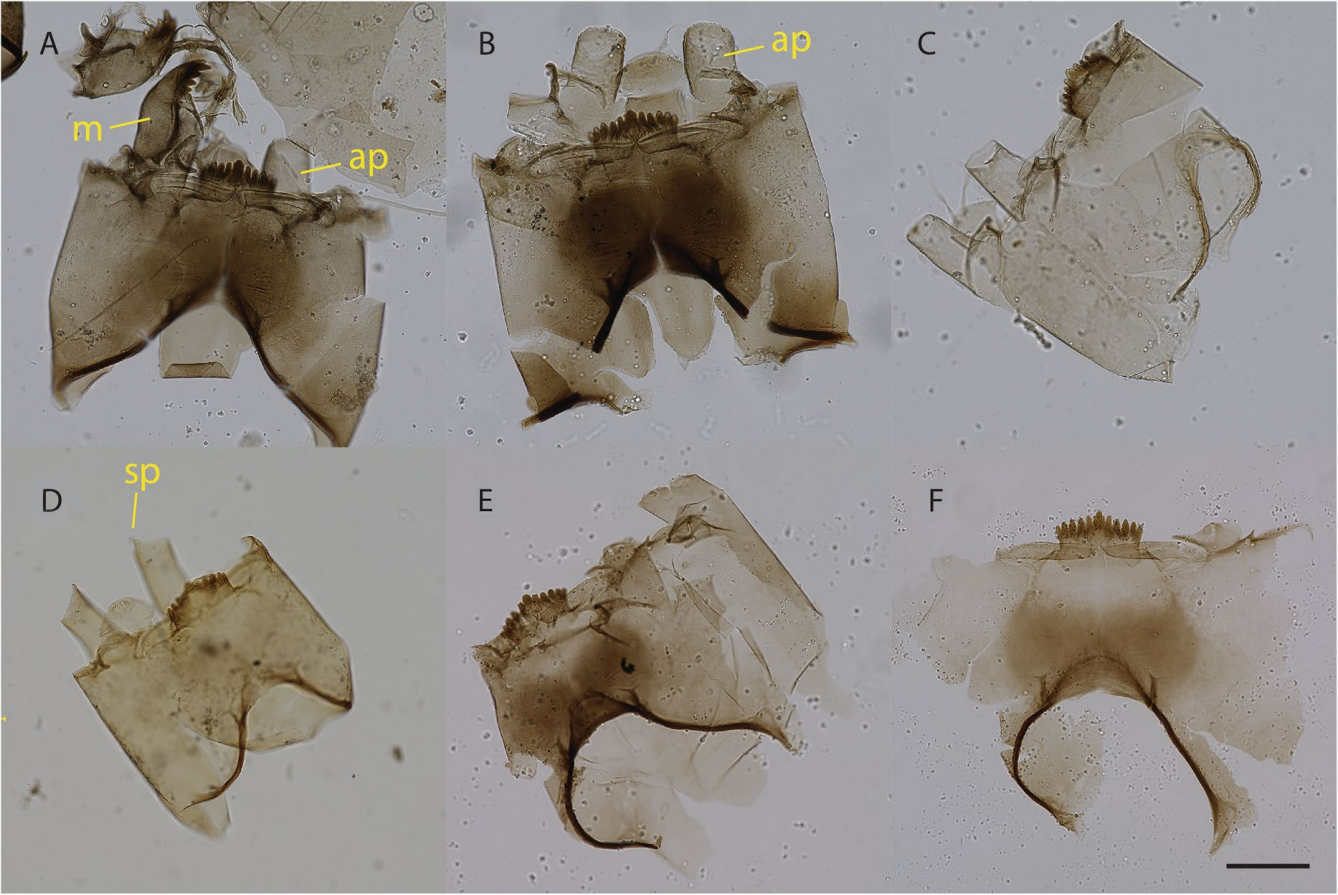
Examples of different morphotypes and levels of identification within the tribe Tanytarsini: **A** specimen identifiable to species morphotype level (*Tanytarsus lugens*-type), in this case based on the morphology of the mandibles (m) and the absence of a spur on the antennal pedestals (ap). **B**–**D** specimens identifiable to an intermediate level, all three can be assigned to broad categories within the genus *Tanytarsus,* **B**, **C** to “*Tanytarsus* no spur” or *Tanytarsus lugens*/*mendax*-type if only these morphotypes without spur are present in a record, and **D** to “*Tanytarsus* blunt spur”, which can, e.g., include specimens of *Tanytarsus pallidicornis*-type (sp indicates the position of the spur on one of the pedestals). **E**, **F** specimens identifiable only to the genus level (*Tanytarsus* undiff.), and not assignable to “*Tanytarsus* no spur” or “*Tanytarsus* blunt spur” since the antennal pedestals are obscured and folded (**E**) or missing (**F**). Not pictured is the lowest-level classification to Tribe level, which should be used for specimens where it is not even clear to which genus within the Tanytarsini they belong

Fossil chironomid head capsules are usually identified to species, species morphotype/species group or genus based on the shape and structure of the mentum and ventromental plates that are firmly attached to the head capsule and present in most specimens. However, the identification of some important subgroups of the Chironomidae, such as the Tanytarsini, relies strongly on additional features such as the morphology of the mandibles and antennal pedestals. These head capsule structures are regularly lost, damaged or obscured in fossil specimens hampering identification. This will for some taxa lead to a situation where some individuals are identified to the most detailed level of identification, whereas others cannot be reliably separated from closely related groups or can only be identified to genus-level or an even coarser level of taxonomic resolution (Fig. 1; Table 1). Depending on the experience of the analyst and the taxa involved, some specimens may also be identified as likely belonging to a certain category, e.g. based on the overall appearance or coloration of the fossil, but without confirmation through structures which clearly associate the specimen with this group. For such tentatively identified specimens it will have to be a conscious decision by the analyst whether they are eventually assigned to the more detailed taxonomic level or not, depending on the confidence with which such an assignment can be made, but also on the research question at hand. However, even if such tentatively assigned specimens are assigned to a species morphotype, there will typically still be specimens identified to several levels of taxonomic detail in the dataset. Figure 1 and Table 1 show, as an example, different taxonomic categories for the Tanytarsini that could be present in a dataset. These could include:

1. Species morphotype level identifications (e.g. *Tanytarsus mendax*-type, *Tanytarsus lugens*-type);
2. Identifications to closely related taxa (e.g. *Tanytarsus mendax*/*lugens*-type or “*Tanytarsus* no spur” as used in some regional chironomid-temperature calibration datasets (Brooks and Birks 2001));
3. Identifications to the genus level (e.g. *Tanytarsus* undiff.);
4. Higher level identifications at the tribe or subtribe level (e.g. Tanytarsini or Tanytarsina) when it is not possible to confidently assign a specimen to the genus *Tanytarsus* or to another genus within the tribe (e.g. *Micropsectra* or *Paratanytarsus*).

**Table 1:** Illustration of the effects of each strategy for dealing with identifications to different levels of taxonomic resolution within the Tanytarsini, based on a theoretical sample of unprocessed counts and six identified taxonomic categories (see text for details)

	*T. lugens*- type	*T. mendax*-type	“*Tanytarsus* no spur”	“*Tanytarsus* blunt spur"	Tanytarsus pallidicornis-type1	*Tanytarsus* undiff	Total counts
Initial counts	10	10	10	10	10	10	60
Combining strategy	0	0	0	0	0	60	60
Retaining strategy	10	10	10	10	10	10	60
Deleting strategy	10	10	0	0	10	0	30
Assigning strategy	18	18	0	0	24	0	60

*Tanytarsus lugens*-type and *Tanytarsus mendax*-type head capsules do not have a spur on the antennal pedestal and, in absence of other diagnostic features such as mandibles, will often be identified to the category “*Tanytarsus* no spur”. In contrast, *Tanytarsus pallidicornis*-type1 have a short blunt spur on the pedestal and in the absence of other diagnostic features will often be identified to the category “*Tanytarsus* blunt spur”

## Strategies for dealing with different levels of taxonomic detail

Intuitively, there are several different ways to deal with the problem that specimens that potentially originate from the same taxon have been identified to different taxonomic levels within the same dataset. Most chironomid analysts have decided on one of the following strategies: The most cautious approach (Procedure 1—“Combining strategy”) is to combine all the identified specimens at the least detailed identification level, e.g. at levels 3 or 4 (i.e. *Tanytarsus* undiff., Tanytarsini or Tanytarsina) in the list provided above). This approach will result in no falsely classified specimens, but it will also result in a significant loss of taxonomic resolution (Table 1). The second option (Procedure 2—“Retaining strategy”) is to keep different levels of identification in the analysed dataset, although this will result in specimens potentially originating from the same species to be classified in different taxonomic units. Procedure 3 (“Deleting strategy”) would be to retain the original different levels of identification, but to delete all identifications above a certain selected level of taxonomic resolution. For instance, all taxa that could not be reliably assigned to species morphotype- or genus-level could be removed from the dataset prior to subsequent analyses (Table 1). The final option (Procedure 4—“Assigning strategy”) is to assign specimens of less well identified categories to the most detailed taxonomic units based on the ratio between specimens that have clearly been identified to this detailed taxonomic level.

## Approaches used to illustrate the effects of the different strategies

**Fig. 2:**
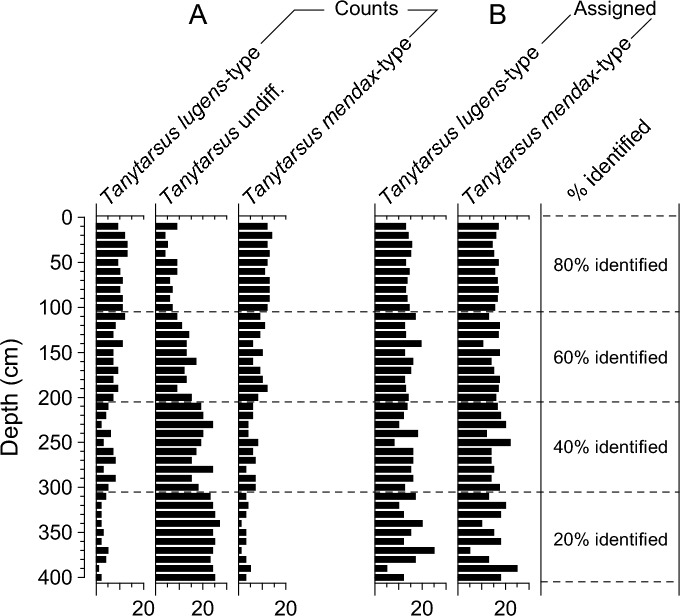
Example of simulated chironomid count data with different percentages of specimens identified to the highest taxonomic level. The example is based on two taxa, *Tanytarsus lugens*-type and *Tanytarsus mendax*-type, that are prescribed to be present in the samples at identical abundances. Random data (**A**) are produced based on a binomial distribution model and assuming that each head capsule has an 80%, 60%, 40% or 20% probability of being identified to the highest taxonomic level (i.e. to *Tanytarsus lugens*- or *Tanytarsus mendax*-type). Specimens not identified to this level are assigned to the category *Tanytarsus* undiff. The two plots on the right (**B**) show the results if head capsules assigned to *Tanytarsus* undiff. are split into the categories *Tanytarsus lugens*-type and *Tanytarsus mendax*-type based on the ratio of specimens identified to these species morphotypes in (**A**) following Procedure 4 (Assigning strategy) outlined in the main manuscript text

All four procedures described above have advantages but also major disadvantages which may influence the data and numerical analyses based on these. In the following section we describe some of these advantages and disadvantages, and we show how the selected procedure may potentially influence subsequent analyses of the chironomid data. To illustrate the effects of the different procedures on chironomid datasets, we discuss how these influence both hypothetical, simulated data and an actual, analysed dataset representing a Lateglacial-to-Holocene chironomid record from Central Europe. The effects of Procedure 4 (Assigning strategy) on datasets were explored by examining two hypothetical examples of counts for assemblages (samples) that exist of 24 headcapsules each belonging to either *Tanytarsus mendax*-type or to *Tanytarsus lugens*-type. These taxa are morphologically similar and can only be reliably separated if mandibles are present, which often is not the case when analysing fossil head capsules. In our first simulated dataset, we prescribed the abundance of each of the taxa to 50% of the assemblage (i.e. 12 counts each), and these prescribed abundances stay identical throughout the 40 simulated samples of a hypothetical record (Fig. 2). Simulated data were produced based on a binomial distribution model and assuming that each head capsule has an 80%, 60%, 40% or 20% probability of being identified to the most detailed taxonomic level (i.e. to *Tanytarsus lugens*- or *Tanytarsus mendax*-type), with specimens that were not identified to this level being assigned to the coarser-resolution taxon *Tanytarsus* undiff. (see supporting online information for more details on the numerical approach used to generate these data). The “Assigning strategy” was subsequently applied to the specimens scored as *Tanytarsus* undiff. morphotype, and they were assigned to either *T. mendax*-type or *T. lugens*-type based on the ratio of specimens identified to the most detailed taxonomic resolution.

**Fig. 3:**
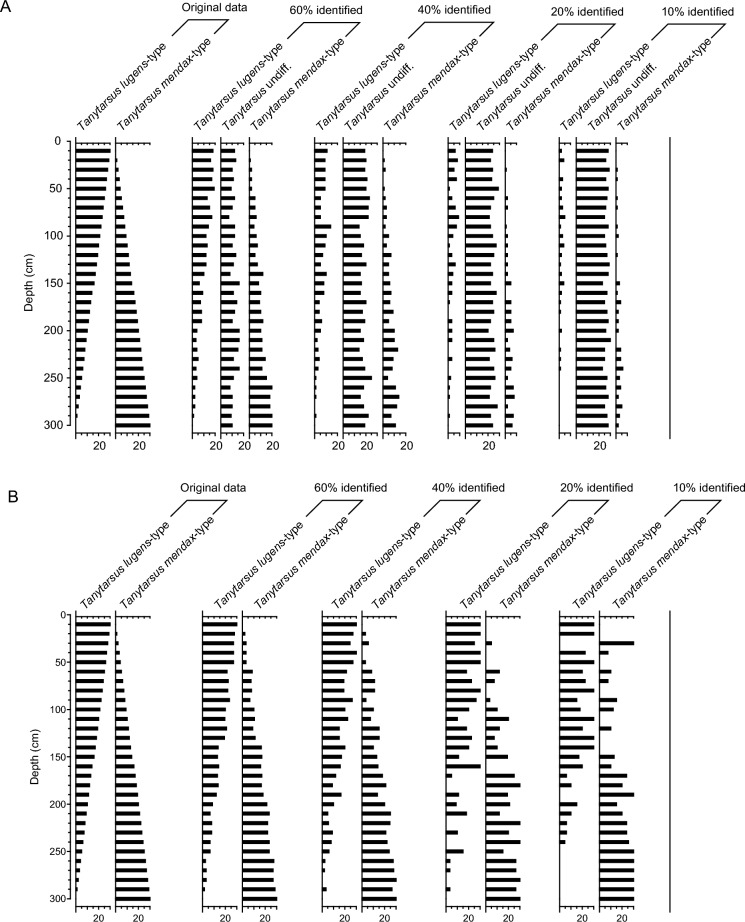
Example of simulated chironomid count data with different percentages of specimens identified to the highest taxonomic resolution and a transition from 100% *Tanytarsus lugens*-type and 0% *Tanytarsus mendax*-type in the uppermost sample to 0% *Tanytarsus lugens*-type and 100% *Tanytarsus mendax*-type in the lowermost sample. **A** shows the number of specimens assigned to the categories *Tanytarsus mendax*-type, *Tanytarsus lugens*-type and *Tanytarsus* undiff. based on simulations with a binomial distribution model and assuming that each head capsule has a 60%, 40%, 20% or 10% probability of being identified to the highest taxonomic level (left to right in the upper panel). **B** shows the results if the head capsules assigned to *Tanytarsus* undiff. in (**A**) are split into the categories *Tanytarsus lugens-* and *Tanytarsus mendax*-type based on the ratio of specimens identified to these species morphotypes following Procedure 4 (Assigning strategy) outlined in the main manuscript text

In our second simulated dataset (Fig. 3), the abundance of *Tanytarsus lugens*-type changed gradually from 100 (24 specimens) to 0%, whereas the abundance of *Tanytarsus mendax*-type changed gradually from 0 to 100%. We again classified specimens to the morphotypes *Tanytarsus mendax*-type, *Tanytarsus lugens*-type and *Tanytarsus* undiff. based on simulations with a binomial distribution model, assuming that each head capsule has a 60%, 40%, 20% or 10% probability of being identified to the highest taxonomic level. Subsequently, specimens classified to the coarser-level *Tanytarsus* undiff. morphotype were assigned to more detailed categories based on the ratio of specimens identified to these species morphotypes (Procedure 4).

**Fig. 4:**
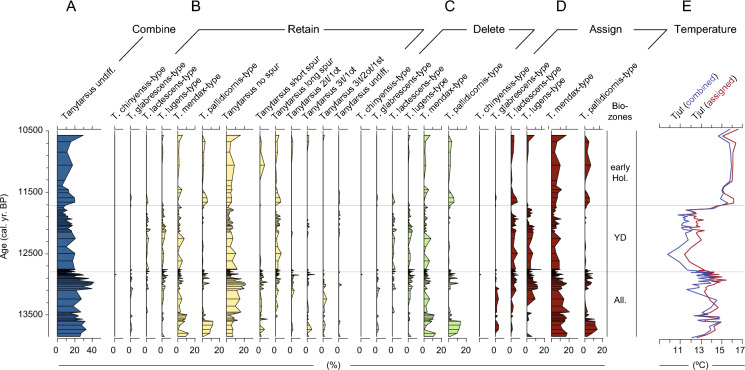
Percent-abundances of *Tanytarsus* taxa in the chironomid record from Lake Hämelsee under each of the four scenarios: **A** Combining procedure; **B** Retaining procedure; **C** Deleting procedure; **D** Assigning procedure. Specimens not identified beyond tribe level (e.g. Tanytarsini undiff.) have been left out of the dataset prior to calculating percent abundances. **E** indicates chironomid-based July air temperatures (Tjul) estimated for the Hämelsee record using a chironomid-temperature transfer function and calibration dataset from Norway and Switzerland and the Combining and Assigning procedure (see text for details). The taxa “*Tanytarsus* no spur”, “*Tanytarsus* short spur” and “*Tanytarsus* long spur” refer to specimens where the mandibles were absent or obscured, and only the characteristics of the antennal pedestal could be used for identification. The taxa “*Tanytarsus* 2it/1ot”, “*Tanytarsus* 3it/1ot” and “*Tanytarsus* 3it/2ot/1st" refer to specimens where the antennal pedestals were absent or obscured, and only the characteristics of the mandible could be used for identification. Abbreviations: it = inner teeth, ot = outer teeth, st = surface teeth. The taxon *Tanytarsus* undiff. featured neither a visible mandible nor an antennal pedestal and could therefore not be identified beyond the genus level. Biozones: Hol. = Holocene; YD = Younger Dryas; All. = Allerød; cal. yr. BP = calibrated ^14^C years BP

The impacts of each of the four procedures on an actual chironomid count dataset were examined for the chironomid record of Lake Hämelsee, Germany (Engels et al. 2022; 2024a, b). This record spans the Late Glacial / Interglacial Transition (LGIT) and we examine the time interval between the Allerød and the Early Holocene (13850–10460 calibrated ^14^C years BP). We applied the strategies of combining, retaining, deleting (here applied to all taxa that are not identified to species morphotype) and assigning to the original count data of Engels et al. (2022; 2024a, b) to illustrate the effects of each of these strategies on a downcore record (Fig. 4). To provide an example of how the selected procedure can influence quantitative analyses we also applied a chironomid-temperature transfer function based on a calibration dataset from Norway and Switzerland to the Hämelsee record (see Heiri et al. 2011 and Supporting Online Information for details on the calibration data, transfer function and numerical procedures used). These calculations show how selecting the Assigning or Combining strategy affects estimates of past changes in mean July air temperatures during the LGIT based on this dataset. Since the calibration dataset was not available as original count data, but only as processed percentage data, it was not possible to explore the effects of the Deleting or Retaining strategy on the results.

## Advantages and disadvantages of the different strategies

The Combining strategy (Procedure 1) where taxa are combined at a low taxonomic level has the clear advantage that it is scientifically rigorous and cautious in the sense that it does not lead to misclassified specimens. However, pooling all specimens at the genus or subtribe level clearly results in a major loss of taxonomic detail and of the potential to make detailed palaeoecological inferences. In our simulated dataset 2 (Fig. 3), the strategy of merging all *Tanytarsus lugens*-type and *Tanytarsus mendax*-type specimens at the level of *Tanytarsus* undiff*.* would obviously fail to detect the clear change from an assemblage dominated by *Tanytarsus lugens*-type to an assemblage dominated by *Tanytarsus mendax*-type.

In actual palaeoecological datasets, several *Tanytarsus* morphotypes and other Tanytarsini such as *Micropsectra* or *Paratanytarsus* may coexist in the same samples. In such samples, the combining strategy would lead to a pooling of specimens at the level of *Tanytarsus* undiff. or even the subtribe Tanytarsina, which in many cases would result in the merging of important indicator species for differing environmental conditions. For example, the development of identification guides for species morphotypes within the Tanytarsini in the 1990s and 2000s has allowed the identification of several species morphotypes typical for relatively cold, oxygen-rich environments (e.g. *Micropsectra radialis*-type, *Tanytarsus lugens*-type, *Paratanytarsus austriacus*-type) or relatively warm environments (e.g. *Tanytarsus glabrescens*-type, *Tanytarsus lactescens*-type, *Tanytarsus pallidicornis*-type2). These taxa often play an important role in detecting responses of chironomid assemblages to eutrophication (Brodersen and Quinlan 2006), to variations in water depth (Luoto 2009), or to climate change (Heiri et al. 2014), and they are important in many chironomid-temperature calibration datasets (e.g. Bakumenko et al. 2024; Suranyi et al. 2025). Figure 4A demonstrates the effects of combining all *Tanytarsus* morphotypes into a single genus-level morphotype for the chironomid record from Lake Hämelsee, Germany. The resulting curve (Fig. 4A) shows overall high abundances (15–40%) throughout the record but as the *Tanytarsus* undiff. morphotype combines taxa with preferences for high temperatures, shallow water depths and relatively high nutrient conditions (e.g. *Tanytarsus pallidicornis*-type) with taxa that prefer cold temperatures, deep water and relatively low nutrient concentrations (e.g. *T. lugens*-type), little palaeoecological information can be derived from such a combined curve. Although combining closely related but poorly separable taxa will still be an option in some situations and datasets, this is clearly not the best strategy when analysts want to examine and interpret their datasets at a maximum taxonomic resolution.

The procedure of retaining multiple levels of taxonomic resolution in the dataset (Retaining strategy, Procedure 2) again has obvious advantages, in this case that the data are presented at the taxonomic resolution they were actually identified at. However, there are two major, perhaps not so apparent disadvantages. First, several taxonomic categories which may contain specimens of the same species are retained in the datasets. A simple example of this may be a record which contains only one Tanytarsina taxon such as *Tanytarsus lugens*-type. When analysing the dataset, specimens originating from *Tanytarsus lugens*-type may be represented by this morphotype, but also by taxa at a coarser taxonomic resolution such as *Tanytarsus* undiff., Tanytarsina or even Tanytarsini, and major changes in the abundance of specimens belonging to *Tanytarsus lugens*-type will also lead to variations in all these categories. As a consequence, the same species or chironomid group may be represented by several taxa in chironomid datasets, potentially amplifying its importance in numerical analyses of the data. Retaining multiple taxa that could reflect the same species would also violate assumptions or prerequisites for the calculation of diversity measures such as Shannon Index or Rarefaction. Second, the preservation state of chironomid assemblages will potentially have a strong influence on the abundances of specimens of the same species that can be identified to the most detailed taxonomic level and those that can only be identified to a coarser taxonomic resolution. In our simulated data, a decrease of the proportion of chironomid remains that can be identified to *Tanytarsus lugens*-type or *Tanytarsus mendax*-type leads, e.g., to an increase in *Tanytarsus* undiff*.* (Figs. 2 and 3). This may lead to apparent changes in species composition of chironomid assemblages in downcore records which only depend on the preservation state of the chironomid fossils (determining the proportion that can be identified to species morphotype level), even in records with no changes in species composition (Fig. 2). Preservation states of chironomid remains can change substantially in some downcore records (e.g. across cold and warm climatic periods, across phases of lake level decrease and partial drying of the coring site). For example, in the Lake Hämelsee record the preservation of chironomids was poor in the varved section of the late Allerød, which resulted in many head capsules missing mandibles and being classified as “*Tanytarsus* no spur” (Fig. 4B). However, this apparent increase in “*Tanytarsus* no spur” is interpreted as an effect of changes in preservation rather than an increase in a particular taxon. The issue of changes in preservation may be particularly relevant for records which cover widely varying depositional environments (e.g. from oxygen rich to oxygen poor conditions or from minerogenic to organic sediment type).

A strategy for dealing with different levels of taxonomic resolution within a sample or dataset that is used in the literature and that is derived from the Retaining strategy is the Deleting strategy (Procedure 3), where specimens that have not been identified to species morphotype are removed from the assemblage prior to subsequent analysis (e.g. Bakumenko et al. 2024). However, the problem of apparent changes in assemblages that are due to preservation rather than actual shifts in species abundance, as described for the Retaining strategy, would also exist when applying the Deleting procedure. If *Tanytarsus lugens*-type and *Tanytarsus mendax*-type would be retained in the dataset (and specimens identified to *Tanytarsus* undiff. deleted), these categories would still be expected to increase or decrease in abundance depending on the proportion of specimens that can be identified to the most detailed taxonomic resolution (Figs. 2 and 3). An additional issue with the “Deleting procedure” stems from the fact that not all chironomid subfamilies and tribes are affected equally by issues of incomplete preservation. For instance, whereas identifications in the tribe Tanytarsini can often be hampered by missing morphological features (e.g. no mandibles preserved), specimens from the Orthocladiinae subfamily are much more rarely affected by such issues. When coarse-level identifications are deleted from a dataset, this could lead to a preferential removal of Tanytarsini over Orthocladiinae, which could be problematic, as even at the subfamily or tribe level these taxa typically represent different nutrient, oxygen or temperature conditions. Finally, deleting observations from a dataset might lead to decreased count sums per sample (Table 1), and might necessitate further laboratory analysis. This would also have been the case in the example of Lake Hämelsee (Fig. 4c). Whereas the removal of specimens that have not been identified to species morphotype and recalculation of the percent-abundances of the remaining taxa did not result in major changes in the shape of each individual curve in the Lake Hämelsee record, overall count sums did reduce as a result of applying the Deleting strategy.

Data processed following the Retaining strategy (Procedure 2) may be very useful for presenting count data, and allowing the reader to assess the level of taxonomic detail that critical taxa have been identified to in different sections of downcore records. However, datasets prepared using the Retaining strategy or the Deleting strategy (Procedure 3) may be less useful for statistical analyses of downcore changes in species assemblages and for visualizing general trends in the data, due to possible artefacts in the data that are the result of the preservation state (and therefore the level of taxonomic detail that most specimens can be identified to).

Finally, the Assigning strategy (Procedure 4) involves the splitting of specimens that have been identified to a less detailed taxonomic resolution to more detailed taxonomic categories. Assignment could be achieved based on the proportion of specimens that can be reliably assigned to these better resolved taxonomic units. For example, in our simulated datasets, this would involve Assigning specimens of the lower-resolution taxonomic category of *Tanytarsus* undiff. into the categories *Tanytarsus lugens*-type or *Tanytarsus mendax*-type based on the ratio of clearly identified *Tanytarsus lugens*-type to clearly identified *Tanytarsus mendax*-type. The Assigning approach clearly makes the assumption that the ratio between identified specimens in the high-resolution taxonomic categories of interest is representative for those fossil remains that could not be identified to the highest taxonomic resolution. It is also clear that the success of this approach strongly depends on the proportion of specimens that could actually be identified to the highest taxonomic resolution. If the number of specimens identified to this level is too low, then the ratio between the identified morphotypes will not provide a very reliable basis for splitting specimens that could only be identified to a coarser taxonomic resolution.

Furthermore, the approach may become very complex if several similar morphotypes can be found within one sample (e.g. if head capsules identified to *Tanytarsus* undiff. need to be split between 4–5 *Tanytarsus* species morphotypes). The approach will obviously break down in situations where sections of the analysed sediment core do not contain any specimens identified to the highest taxonomic level. However, the Assigning approach has, in principle, the potential to correct for different levels of preservation within sediment cores and may estimate similarly high abundances of species morphotypes in samples with very different proportions of specimens identified to the highest taxonomic levels.

Our simulated count data show how the Assigning procedure would be expected to affect estimated counts in samples with different levels of head capsules identified to the most detailed taxonomic resolution. If the samples with identical abundances of *Tanytarsus lugens*-type and *Tanytarsus mendax*-type but different proportions of specimens identified to the highest taxonomic resolution are examined, it becomes clear that the Assigning procedure leads to an increasing variability in the estimated abundances of *Tanytarsus lugens*-type and *Tanytarsus mendax*-type with decreasing preservation of fossil remains (Fig. 2). However, on average the Assigning approach is able to reconstruct the true (in this case prescribed) abundances with no evidence of a systematic error in the data. This suggests that even in very variable records, averaged or smoothed values may provide realistic estimates of the abundances of the species morphotypes. When simulated data with clear trends in the prescribed abundances of *Tanytarsus lugens*-type and *Tanytarsus mendax*-type are examined (Fig. 3), it becomes apparent that the approach seems to be successful at reconstructing the trends even in situations where only a low proportion (10–40%) of specimens have been identified to the highest taxonomic level (Fig. 3B). The percent-abundance values after assigning again become highly variable at the lowest proportions of specimens identified to the highest resolution (e.g. at 10%), but smoothed records would represent the transition from assemblages dominated by *Tanytarsus lugens*-type to those dominated by *Tanytarsus mendax*-type relatively well, even in these situations.

In the Lake Hämelsee dataset the Assigning procedure reduced the original number of 13 morphotypes across all levels of identification (e.g. as included in the Retaining strategy; Fig. 4B) to six species-level morphotypes (Fig. 4D). In this case study, the major advantage of the Assigning approach over e.g. the Combining or Retaining strategies is that the resulting curves provide meaningful palaeoecological information about past lake ecosystem dynamics. For instance, the warm-indicating taxon *T. pallidicornis*-type disappears from the assemblages at the onset of the Younger Dryas. This change is clearly visible in the Assigning strategy plot (Fig. 4D) but less obvious in the Retaining and Deleting strategy plots (Fig. 4B, C), and this information is completely lost when the Combining strategy is applied (Fig. 4A).

When variations in mean July air temperatures are estimated from the Hämelsee record using the chironomid-temperature transfer function from Norway and Switzerland using the Combining and Assigning strategy (Fig. 4E), both approaches provide a very similar reconstruction of past temperature changes during the LGIT at this locality. Nevertheless, minor systematic differences between the two reconstructions are apparent, e.g. during the Younger Dryas period when the Combining strategy results in cooler reconstructed temperatures than the Assigning strategy. In the case of Hämelsee, these differences are clearly smaller than the prediction error of the transfer function which has been estimated to 1.40 °C (Heiri et al. 2011). However, the Deleting or Retaining strategy could not be applied when reconstructing temperatures from the Hämelsee record, since the original count data of the applied transfer function was not available at a level of detail which would have allowed the application of these strategies. Furthermore, offsets between the different strategies may obviously be more critical for chironomid records with a higher overall abundance of Tanytarsini head capsules than observed in the Hämelsee record or for different lake types, environmental reconstructions or quantitative analyses. Future publication and archiving of calibration datasets at maximum taxonomic resolution and before processing of count data using the Combining, Retaining, Deleting or Assigning strategy would be essential to allow a more rigorous assessment of the effects of these strategies on quantitative paleoecological reconstructions than was possible in this study.

## Application of the assigning strategy

Of the four procedures described above, the assignment of less well identified specimens to categories that represent a higher taxonomic resolution (Procedure 4) involves the highest number of decisions on the exact numerical procedure used and may be the least familiar to researchers new to fossil chironomid research. We therefore briefly describe one possible way of assigning head capsules to taxonomically better resolved categories to illustrate some of the decisions that analysts should consider and standardize if they decide to use this approach.

*Step 1* Decide on the taxonomic categories that should be assigned to categories identified to a higher taxonomic resolution and categories that represent a too coarse taxonomic resolution to be treated this way. For example, Tanytarsini taxa that could be assigned to other, more detailed taxonomic units could include categories that include morphologically similar but not separated species morphotypes (e.g. *Tanytarsus lugens*/*mendax*-type, *Tanytarsus glabrescens*/*lactescens*-type), broader categories including several *Tanytarsus* morphotypes (such as the colloquial “*Tanytarsus* no spur” or “*Tanytarsus* blunt spur”) or genus level identifications (e.g. *Tanytarsus* undiff., *Micropsectra* undiff.). Categories not to be merged could include tribe, subtribe, or family level identifications (e.g. Tanytarsina, Tanytarsini, Chironominae or Chironomidae). The latter would be considered as unidentified chironomid remains and would then not be included in further quantitative analyses of the results. However, analysts might consider including these unidentified counts when calculating overall concentrations of chironomid remains (typically expressed as number of head capsules per weight or volumetric unit).

*Step 2* Resolve categories that are tentatively associated with a particular morphotype (e.g. *Tanytarsus* c.f. *lugens*-type, *Tanytarsus* nr. *mendax*-type or similar designations). Analysts should consider whether the available taxonomic information is sufficient to merge these with the tentatively associated morphotypes or whether they should be pooled with categories at a coarser taxonomic resolution (in our case, e.g. *Tanytarsus lugens/mendax*-type, “*Tanytarsus* no spur” or *Tanytarsus* undiff.).

*Step 3* Assign specimens in coarse-resolution categories that contain morphologically similar morphotypes to the categories identified to the highest taxonomic resolution based on the ratio of identified specimens in these higher-resolution categories. For the Tanytarsini this could, e.g., include the assigning of specimens identified to *Tanytarsus lugens/mendax*-type or *Tanytarsus glabrescens/lactescens*-type to the categories *Tanytarsus lugens*-type and *Tanytarsus mendax*-type or *Tanytarsus glabrescens*-type and *Tanytarsus lactescens*-type, respectively. This step includes the calculation of the ratio between specimens identified to the highest taxonomic resolution on a sample-by-sample basis, splitting the specimens identified to the lower taxonomic resolution based on this ratio, and then assigning these split counts to the corresponding category that represents the highest taxonomic resolution (e.g. specimens identified to *Tanytarsus lugens/mendax*-type would be split according to the ratio of *Tanytarsus lugens*-type to *Tanytarsus mendax*-type in the same sample and merged with these categories). In theory, assigning can be done with data rounded to single (or halves of) specimens or it can be based on the unrounded ratios. For count data of biotic proxies the former seems more logical as this will result in merged data that still represent full (or half) counts, and not fractions of counts. If the rounding approach is selected, the Assigning procedure ideally also includes a standardized procedure (a “tie-breaker”) in case the analyst cannot decide based on the ratio into which category an individual specimen belongs. For example, in such situations the doubtful specimens can then consistently be merged with the more abundant taxon in the overall dataset or in adjacent samples in the record.

In some samples, particularly in records with low chironomid concentrations and poor preservation states, the situation may occur that no specimens are identified to the highest taxonomic resolution. For example, some samples may contain specimens identified to *Tanytarsus lugens/mendax*-type but none clearly determined to be *Tanytarsus lugens*-type or *Tanytarsus mendax*-type. Analysts should consider how to deal with this situation consciously and in a consistent way within datasets. Solutions may involve deleting the sample in question from the dataset, merging adjacent samples to reach higher count sums for the taxa involved, or splitting of specimens identified to the coarser taxonomic unit based on the ratio of identified specimens in adjacent samples within the same stratigraphic unit.

*Step 4* Once specimens that could not be separated between morphologically very similar species morphotypes have been split and assigned to the categories with the highest taxonomic resolution (e.g. *Tanytarsus lugens/mendax*-type have been assigned to *Tanytarsus lugens*-type or *Tanytarsus mendax*-type), the analyst should consider whether specimens identified to even coarser taxonomic units can also be split among the species morphotypes using the procedure outlined in Step 3, above. For example, specimens only identified to the level of genus or to categories including several species morphotypes (e.g. “*Tanytarsus* blunt spur”) could also be assigned to species morphotypes of this genus in the sample based on the relationship between specimens classified to the highest taxonomic resolution (e.g. in our example shown in Table 1 specimens identified to *Tanytarsus* undiff. could be assigned to *Tanytarsus mendax*-type, *Tanytarsus lugens*-type or *Tanytarsus pallidicornis*-type based on this procedure). As indicated above, at some point these categories will probably become too coarse to allow a useful assignment to better identified categories (e.g. at the level of subtribe, tribe or subfamily).

*Step 5* At the end of the procedure analysts are recommended to consider briefly the success of this approach for assigning specimens identified to coarser taxonomic levels to categories at higher taxonomic resolution. Points of consideration could include whether there are sections of their record or dataset which may be problematic, whether this approach is really necessary for the research question at hand or whether merging of different categories at different taxonomic levels is a useful alternative.

Table 1 provides a theoretical example of how the Assigning strategy can be applied to a sample with a considerable number of head capsules not identified to species morphotype, the highest level of taxonomic detail that Tanytarsini head capsules are usually identified to. Head capsules identified to belong to a subgroup within the genus *Tanytarsus* (“*Tanytarsus* no spur”, “*Tanytarsus* blunt spur”) are first assigned to species morphotypes based on the proportion of head capsules identified to these morphotypes in the sample. As a consequence, “*Tanytarsus* blunt spur” is merged with *Tanytarsus pallidicornis*-type, as this is the only species morphotype with a blunt spur, and “*Tanytarsus* no spur” is split equally between *Tanytarsus lugens*-type and *Tanytarsus mendax*-type, as these two morphotypes without spur are present at equal abundances. In a second step, head capsules identified only to genus level (*Tanytarsus* undiff.) are then split between the three remaining categories in the sample, *Tanytarsus lugens*-type, *Tanytarsus mendax*-type and *Tanytarsus pallidicornis*-type, based on the ratio of head capsules already assigned to these morphotypes (in our example 15: 15: 20). This results in a total 18 specimens that are assigned to *Tanytarsus lugens-type*, 18 to *Tanytarsus mendax*-type and 24 to *Tanytarsus pallidornis*-type, respectively.

## Recommendations

As the examples and considerations above show, all the discussed ways for dealing with different levels of identification for specimens that may originate from the same species or species morphotypes have their own advantages and disadvantages. We therefore strongly believe that there is no one optimal way to deal with this issue, but that analysts will have to determine the best solution to this problem on a case-by-case basis. They should consider the type and quality of data they are working with, as well as the overall aims of their research project. Pooling of taxa into coarser taxonomic categories may be the simplest and most appropriate approach when identification to species or species-morphotype level is not necessary to achieve the project aims. Retaining several levels of taxonomic detail in the data may be useful for visualizing datasets and demonstrating to readers changes in taxonomic resolution and the amount of specimens identified to the highest taxonomic detail versus less well identified specimens across records. Deleting certain taxa could be appropriate if e.g. a project focusses on biodiversity metrics or on species distributions, or if the level of identification is so coarse (e.g. tribe or subfamily level) that the morphotype becomes meaningless from a palaeoecological point of view. Assigning less well identified specimens to categories based on the proportion of well-identified specimens in datasets may be appropriate when reaching the highest level of taxonomic resolution and information on important indicator taxa that are only apparent at this level of identification are essential for the project. This approach may also be appropriate when new datasets are developed that will eventually be merged with or numerically compared to other datasets at this high level of taxonomic detail. In cases where assigning less-well identified specimens to categories identified to the highest taxonomic resolution may be problematic, or the effects of merging on the results remain unclear, analysts may consider one of the following strategies: Results of ecological or numerical analyses with the taxa merged (Procedure 1, Combining strategy) can be compared with the results when taxa representing a coarser taxonomic resolution are assigned to better identified taxa based on proportions (Procedure 4, Assigning strategy). In our experience, results based on these two approaches are very similar for some datasets, as for example in our temperature reconstruction from the Hämelsee record (Fig. 4E), in which case the decision does not really impact the interpretations much. In other datasets, the results may differ significantly, and this can then be taken into account when interpreting the data. Alternatively, the count sum of critical samples may be increased (e.g. by analysing more sediment or merging adjacent samples) which will often lead to a higher count of specimens identified to the highest taxonomic resolution and also a better basis for assigning head capsules identified at a lower taxonomic resolution to more detailed taxonomic categories. Finally, for data where assignment (i.e. Procedure 4) leads to a very high between-sample variability in downcore records in the raw abundance data, and in numerical interpretations obtained from these (e.g. reconstructed values of environmental variables such as temperature), analysts may consider reducing this variability by smoothing the results e.g. with moving averaging, GAM or loess smoothers, as our simulations suggest that the procedure mainly adds variability in the abundance data and does not necessarily result in systematic biases favouring one taxon over another.

Based on the considerations above we can also formulate clear recommendations for the community of fossil chironomid researchers on how to deal with this issue in future work. First, we believe it is important that the community becomes aware of this issue and that different solutions and strategies exist to deal with it. Particularly, we believe it is important to also inform younger researchers and newcomers to the field, and raise their awareness that deciding for or against the strategies described above can potentially have an important influence on the results of ecological and numerical analyses of chironomid count data. Second, we believe it is important that authors try to at least briefly declare in their publications how this problem was dealt with. This can take the form of a very brief statement indicating which procedure (Combine, Retain, Delete or Assign), or combination of procedures, has been selected to address the issue of multi-level taxonomic resolution in their dataset. Finally, where the format of the journal and research publications allows, we recommend analysts to consider archiving not only their processed datasets as supporting online information, but also the original datasets of counts before merging taxa (or samples) or splitting of less well identified categories into taxa at the highest taxonomic resolution. This will allow other researchers to understand the level of taxonomic detail in the original count data and how these were converted to data suitable for ecological and numerical analyses.

Detailed reporting and archiving of chironomid datasets before merging or splitting individual taxa would also allow a more rigorous assessment of how the different strategies affect quantitative analyses based on microfossil datasets. For example, at present it remains unclear how the performance of quantitative inference models or reconstructions based on transfer functions or ordinations are affected by selecting the Retaining versus the Assigning strategy, or how the loss of taxonomic resolution associated with the Combining strategy influences the results of quantitative analyses. At the moment, decisions taken during preprocessing of published chironomid—environment calibration datasets strongly limit the options available for analysts that intend to develop quantitative reconstructions based on chironomid datasets, since it seems intuitively clear that downcore records should be treated with the same data processing strategy as the calibration dataset and transfer function that are applied for developing such reconstructions. However, without the original count data, calibration datasets processed with the Delete strategy cannot be processed with the Assign strategy or vice-versa. Similarly, calibration data processed with the Combine strategy cannot be reverted to the original count data or processed with the Delete or Assign strategy. In contrast, datasets processed with the Retain or Assign strategy can still be processed with the Combine strategy, whereas this is not possible for data treated with the Delete strategy, as taxonomic categories not identified to the most detailed taxonomic resolution have already been deleted from such datasets. For example, for the chironomid-based temperature reconstruction from the Hämelsee record, the case study we discuss in this publication (Fig. 4E), we were able to explore the effects of the Combine and Assign strategy on the results, but not the effects of the Delete or Retain strategy. If the applied calibration dataset and transfer function would have been archived and available as original count data, we would have been able to process calibration dataset and downcore record identically for all four strategies, enabling us to implement and explore the Retaining or Deleting strategies as well. More extensive numerical experiments examining the four different data processing strategies would also be necessary to explore the effects of more complex approaches than we discuss here on reconstruction results, such as, e.g., the application of different data processing strategies to different sections of the same downcore chironomid record, depending on the quality and preservation state of subfossil remains or the taxonomic composition of different sedimentary units.

Although this study focussed on examples from fossil chironomid research, we believe the considerations and approaches we describe are also relevant to researchers working with other microfossil groups commonly found in lake sediments, such as cladocerans, diatoms, ostracods, or pollen. In datasets resulting from such analyses, taxa representing different levels of taxonomic detail may also have been identified during microscopic analysis, resulting in similar issues as we describe here for fossil chironomid datasets. We therefore hope that our work also contributes to discussions of the effects of merging or splitting of count data, and possibly to standardizing these approaches in these fields of research.

## Supplementary Information

Below is the link to the electronic supplementary material.

Supplementary file1 (DOCX 18 KB)

## Data Availability

Simulated count data are presented in Figs. 2 and 3 and the procedure used to produce them is described in the supporting information. The source of the dataset presented in Fig. 4 is provided in the manuscript text.
