# Supplementary material for: Combine, assign or delete? How to resolve different levels of taxonomic identification in chironomid datasets
Source: J Paleolimnol. 2026 Mar 30;74(2):6. doi: 10.1007/s10933-026-00387-1 (PMC13035590; doi:10.1007/s10933-026-00387-1)
Supplement: Supplementary file 1 — Supplementary file1 (DOCX 18 KB) [file 10933_2026_387_MOESM1_ESM.docx]

**Supporting online information:**

Heiri O., Engels S., Combine, assign or delete? How to resolve different levels of taxonomic identification in chironomid datasets. Journal of Paleolimnology.

**Procedure for producing the simulated count data in Figures 2-3:**

The simulated count data shown in Figures 2-3 have been produced with MS Excel using the function =BINOM.INV(A, p, RAND()), where A indicates the prescribed count of *Tanytarsus lugens*-type or *Tanytarsus mendax*-type in a given sample, and p the probability that a specimen of this morphotype cannot be identified to the highest taxonomic level and has to be assigned to the category *Tanytarsus* instead. In Figure 1 the function was run for 10 replicate samples each with a p value of 0.2, 0.4, 0.6 and 0.8, representing a prescribed probability of 80%, 60%, 40% and 20% that the specimens can be identified to the species morphotype (one simulation per sample shown in the figure). For Figure 2 the function was run with a p value of 0.4, 0.6, 0.8 and 0.9, representing a prescribed probability of 60%, 40%, 20% and 10% that the specimens can be identified to the species morphotype (one simulation per sample shown in the figure).

**Procedure for developing temperature reconstructions in Figure 4:**

To illustrate the effects of the four strategies discussed in this manuscript on a quantitative temperature reconstruction, we applied the Combine and Assign strategies to both the fossil dataset from Hämelsee (Engels et al. 2022; 2024a, b) as well as to the Swiss-Norwegian chironomid-temperature calibration dataset (Heiri et al., 2011). The training set data as published on NOAA has already undergone a strategy of deletion of specimens identified at the tribe- or subfamily level (e.g. Orthocladiinae unid.) and subsequent assigning of specimens identified to genus but not morphotype level (e.g. *Tanytarsus* spp.). This existing processing precludes to test the effects of the Retain and Delete strategies on the Hämelsee temperature reconstruction.

To align the fossil dataset with the training set taxonomy, we first removed the specimens identified at tribe or subfamily level from the fossil dataset before applying the Combine and the Assign strategies. Whereas this manuscript focusses on the example of incomplete identifications of *Tanytarsus*-specimens, the Combine and the Assign strategies were applied to the complete chironomid assemblages from the Hämelsee record. This means that incomplete identifications of genera other than *Tanytarsus* (e.g. *Chironomus* spp., *Corynoneura* spp.) were also Combined or Assigned as described above, both in the fossil and in the training set data.

Only 120 specimens of the total 539.5 hcs belonging to the genus *Paratanytarsus* could be assigned to morphotype level. As this number was considered too low to allow a meaningful Assign strategy, we amalgamated the taxa *P. austriacus*-type and *P. penicillatus*-type into the generic taxon *Paratanytarsus* spp., both in the fossil and in the modern dataset. This combining strategy was used in both reconstructions presented here (Combine and Assign) and is in line with the approach used in the original publication presenting the chironomid-inferred climate reconstructions (Engels et al., 2022). It should be noted that the numbers of specimens in the latter categories were much lower than those in the *Tanytarsus* genus. Specimens present in the Hämelsee record that were identified to a morphotype level that was not present in the calibration dataset (e.g. *Micropsectra junci*-type) were retained in the dataset and e.g. used for percentage calculation.

For each strategy (Combine, Assign), we applied the same training set and model characteristics as originally published in Heiri et al. (2011) regarding outlier exclusion, data transformation and model selection. The applied chironomid-temperature transfer function after the taxon amalgamations described above was characterized by a bootstrapped (9999 cycles) coefficient of determination (r^2^) of 0.86 and 0.83, a root mean square error of prediction (RMSEP) of 1.46 and 1.65 (°C), and a mean value for the sample-specific estimated standard errors of prediction (eSEP) of 1.43 and 1.60°C for the Assigning and Combining strategy, respectively. All calculations were done with the program C2 (Juggins, 2007) version 1.8.0.

**References**

Engels S, Lane CS, Haliuc A, Hoek WZ, Muschitiello F, Baneschi I, Bouwman A, Bronk Ramsey C, Collins J, de Bruijn R, Heiri O, Hubay K, Jones G, Laug A, Merkt J, Müller M, Peters T, Peterse F, Staff R, ter Schure A, Turner F, van den Bos V, Wagner-Cremer F (2022) Synchronous vegetation response to the last glacial-interglacial transition in northwest Europe. Commun Earth Environ 3: 130. https://doi.org/10.1038/s43247-022-00457-y

Engels S, Lane CS, Sachse D, Hoek WZ, Baneschi I, Bouwman A, Brogan E, Bronk Ramsey C, Collins J, de Bruijn R, Haliuc A, Heiri O, Hubay K, Jones G, Jones V, Laug A, Merkt J, Muschitiello F, Müller M, Peters T, Peterse F, Pueschel A, Staff R, ter Schure A, Turner F, van den Bos V, Wagner-Cremer F (2024a) Biodiversity responses to Lateglacial climate change in the subdecadally-resolved record of Lake Hämelsee (Germany). Quat Sci Rev 331: 108634. https://doi.org/10.1016/j.quascirev.2024.108634

Engels S, Lane CS, Hoek WZ, Baneschi I, Bouwman A, Brogan E, Bronk Ramsey C, Collins JA, de Bruijn R, Haliuc A, Heiri O, Hubay K, Jones G, Jones V, Laug A, Merkt J, Muschitiello F, Muller M, Peters T, Peterse F, Pueschel A, Staff RA, ter Schure A, van den Bos V, Wagner-Cremer F (2024b) Chironomidae whole or half head capsules counts in sediment core Haem13 from lake Hämelsee. https://doi.org/10.1594/PANGAEA.964523

Heiri O, Brooks SJ, Birks HJB, Lotter AF (2011). A 274-lake calibration data-set and inference model for chironomid-based summer air temperature reconstruction in Europe. Quat Sci Rev 30: 3445-3456. https://doi.org/10.1016/j.quascirev.2011.09.006

Juggins, S., 2007. C2 Version 1.5 User Guide. Software for Ecological and Palaeoecological Data Analysis and Visualisation. Newcastle University, Newcastle upon Tyne. https://www.staff.ncl.ac.uk/stephen.juggins/software/code/C2.pdf
